# The COVID-19 Driving Force: How It Shaped the Evidence of Non-Invasive Respiratory Support

**DOI:** 10.3390/jcm12103486

**Published:** 2023-05-16

**Authors:** Yorschua Jalil, Martina Ferioli, Martin Dres

**Affiliations:** 1 INSERM, UMRS1158 Neurophysiologie Respiratoire Expérimentale et Clinique, Sorbonne Université, 75006 Paris, France; 2 Service de Médecine Intensive–Réanimation (Département “R3S”), AP-HP. Sorbonne Université, Hôpital Pitié-Salpêtrière, 75013 Paris, France; 3 Departamento de Medicina Intensiva, Facultad de Medicina, Pontificia Universidad Católica de Chile, Santiago 8331150, Chile; 4 Departamento de Ciencias de la Salud, Carrera de Kinesiología, Facultad de Medicina, Pontificia Universidad Católica de Chile, Santiago 8331150, Chile; 5 Respiratory and Critical Care Unit, IRCCS Azienda Ospedaliero-Universitaria di Bologna, 40138 Bologna, Italy; 6 Department of Clinical, Integrated and Experimental Medicine (DIMES), Alma Mater Studiorum University of Bologna, 40136 Bologna, Italy

**Keywords:** COVID-19, coronavirus, critical care, non-invasive ventilation, CPAP, high-flow nasal cannula, high-flow nasal oxygen, mechanical ventilation, acute respiratory failure

## Abstract

During the COVID-19 pandemic, the use of non-invasive respiratory support (NIRS) became crucial in treating patients with acute hypoxemic respiratory failure. Despite the fear of viral aerosolization, non-invasive respiratory support has gained attention as a way to alleviate ICU overcrowding and reduce the risks associated with intubation. The COVID-19 pandemic has led to an unprecedented increased demand for research, resulting in numerous publications on observational studies, clinical trials, reviews, and meta-analyses in the past three years. This comprehensive narrative overview describes the physiological rationale, pre-COVID-19 evidence, and results of observational studies and randomized control trials regarding the use of high-flow nasal oxygen, non-invasive mechanical ventilation, and continuous positive airway pressure in adult patients with COVID-19 and associated acute hypoxemic respiratory failure. The review also highlights the significance of guidelines and recommendations provided by international societies and the need for further well-designed research to determine the optimal use of NIRS in treating this population.

## 1. Introduction

Since the onset of the COVID-19 pandemic in January 2020, there has been a rise in acute respiratory distress syndrome (ARDS), a well-recognized entity and one of the virus’ main life-threatening complications [1,2]. Hospitals and intensive care units (ICU) have been overwhelmed by rising cases of ARDS day after day. Non-invasive respiratory support (NIRS), which comprises non-invasive ventilation (NIV), continuous positive airway pressure (CPAP), and high-flow nasal oxygen (HFNO), has been widely used to support patients with COVID-19 [3]. The benefits of HFNO in hypoxemic acute respiratory failure have been established prior to the COVID-19 era, while the use of NIV and CPAP in de novo acute respiratory failure was still being debated [4], possibly explaining why clinicians were reluctant to use them in the early phase of the outbreak.

Interest in NIRS grew as ICU resources became limited. They were one of the first-line strategies to avoid the risk of ICU overcrowding, as they could also be used in emergency departments, monitored beds, and sometimes general wards. The high mortality observed in patients undergoing invasive mechanical ventilation drew even more attention to NIRS, which avoids the risks and complications associated with intubation and invasive mechanical ventilation [5].

NIRS was a frequent therapeutic choice for patients with COVID-19-related acute respiratory failure, particularly those that requested “no intubation” [6] and in those with major risk factors for developing severe respiratory failure due to COVID-19, such as older age, severe comorbidity, and frailty [7,8]. On the other hand, NIRS use was limited by viral aerosolization fear, potentially infecting caregivers, risks associated with delaying intubation, and potentially worsening primary lung injuries, a process referred to as patient self-inflicted lung injury (P-SILI) [9]. Over the past three years, a significant amount of the literature has reviewed the concerns and clinical recommendations surrounding the use of NIRS in COVID-19 patients, positioning them as feasible strategies able to delay the need for tracheal intubation and reduce mortality [10,11,12]. However, the impact of COVID-19 on the evidence regarding NIRS has not been fully addressed. In this narrative review, we aim to chronologically summarize the research on the use of NIRS for managing acute COVID-19-related respiratory failure, highlighting the evolution of evidence over the years, and how COVID-19 has shaped it.

## 2. Materials and Methods

We conducted a review of the literature using the PubMed and Google Scholar databases to provide an up-to-date overview of the research on NIRS during the early pandemic (2019–2022). Studies were included if they focused on evidence from the pre-COVID-19 era regarding the physiology associated with the use of NIRS (NIV, CPAP, and HFNO) and its rationale for its use for treating patients that develop acute respiratory failure due to COVID-19. The results are presented as a narrative review, where the selection of content is based on the author’s experience, the study type, and the date of publication, including observational studies, clinical trials, reviews, and published guidelines. The evidence and clinical recommendations are summarized in three main sections: physiology and pre-COVID-19 era, observational studies, and clinical trials.

## 3. High-Flow Nasal Oxygen

### 3.1. First Act: The Physiologic Effects and HFNO Development in the Pre-COVID-19 Era

HFNO is a non-invasive open-circuit system for respiratory support that provides heated and humidified air at high flows (up to 80 L/min) through nasal cannulae inserted in the nostrils and provides an inspiratory fraction of oxygen (FiO_2_) ranging from 21% to 100% [13]. HFNO was first developed in the early 2000s to support preterm infants with apnea or parenchymal lung disease, with the objective of providing high oxygen flow rates while avoiding the discomfort of tight CPAP face masks [14,15,16]. Over the past two decades, the use of HFNO has spread from pediatric patients with acute respiratory failure, particularly in the treatment of bronchiolitis [17,18,19,20], to adult populations [21,22,23]. HFNO has therefore become one of the most frequently used NIRS for treating or preventing acute hypoxemic respiratory failure [24].

HFNO provides several benefits in terms of physiology and clinical outcomes [25]. It provides a more stable FiO_2_ than conventional oxygen therapy, matching the patient’s inspiratory flow and reducing the amount of room air mixture. The constant high air flow generates a low level of positive end-expiratory pressure (PEEP) around 2–4 cmH_2_O (if the mouth is closed). HFNO also prevents airway dryness and mucociliary dysfunction which preserves secretion clearance [14,26,27]. Another benefit of HFNO is the continuous washout of carbon dioxide from the anatomical dead space which creates an oxygen reservoir in the upper airways [28]. Physiological studies have shown that the use of HFNO in acute hypoxemic respiratory failure patients is associated with improved key physiological parameters, such as gas exchange, respiratory rate and effort, lung volume, dynamic compliance, and transpulmonary pressures [25,29]. The physiological benefits, the relative simplicity of its use (HFNO devices have, essentially, three settings: FiO_2_, flow, and temperature), and the comfort provided to patients have contributed to the increased use of HFNO in clinical practice prior to COVID-19.

**Figure 1:**
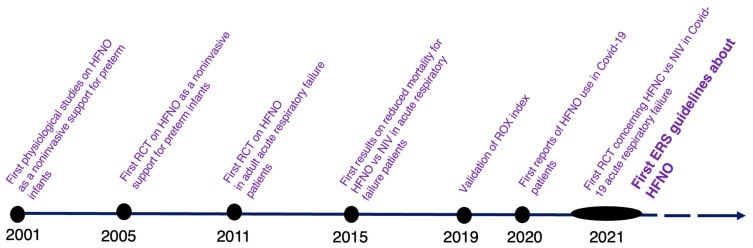
Timeline of HFNO evidence. 2001: Courtney et al. [32], 2005: Capasso et al. [33], 2011: Parke et al. [30], 2015: Frat et al. [17], 2019: Roca et al [34], 2020: Wang et al. [35], 2021: Grieco et al. [36], Oczkowski et al [31].

Between 2011, the year of the first randomized controlled trial (RCT) conducted on adult patients with acute respiratory failure [30], and 2021, when the first version of European respiratory society (ERS) guidelines on HFNO was published (Oczkowski et al. [31]), abundant research has discussed the use of HFNO in various acute respiratory failure scenarios (Figure 1). HFNO has become the first-line treatment for acute hypoxemic respiratory failure when conventional oxygen therapy is insufficient, and intubation is not immediately indicated.

One of the studies that changed the history of HFNO before the COVID-19 pandemic was FLORALI (high-flow oxygen through nasal cannula in acute hypoxemic respiratory failure), a multicenter, open-label trial that evaluated the use of HFNO versus standard-oxygen and NIV in 310 non-hypercapnic patients with a ratio of arterial oxygen partial pressure to fractional inspired oxygen (PaO_2_/FiO_2_) of 300 or less [17]. No statistical difference was found in the primary outcome: intubation rate within 28 days after randomization, but in the subgroup of patients with a PaO_2_/FiO_2_ of 200 mmHg or less, the HFNO group showed a significantly lower intubation rate compared to the other two groups. Despite the enthusiasm generated by the FLORALI trial, the results from other studies comparing HFNO to NIV remained conflicting and strictly dependent on the selected population [16,37,38,39]. However, the concern that HFNO failure may lead to delayed intubation and worsen clinical outcomes in acute respiratory failure patients dampened the enthusiasm [40,41]. The ROX index (ratio of oxygen saturation to fraction of inspired oxygen to respiratory rate) was developed to better identify patients at high risk of intubation during treatment with HFNO [34]. The ROX index provides a non-invasive and real-time measure of a patient’s oxygenation status and has been validated in a cohort of patients with acute hypoxemic respiratory failure [34]. Studies have reported that a ROX index of 4.88 or higher measured at 2, 6, and 12 h is associated with success in HFNO treatment [34]. In conclusion, the ROX index is a useful tool in the management of patients assisted with HFNO and can help identify those at high risk of intubation. 

### 3.2. Second Act: The Observational Cohort Studies in COVID-19 Era—The HFNO Challenge

**Figure 2:**
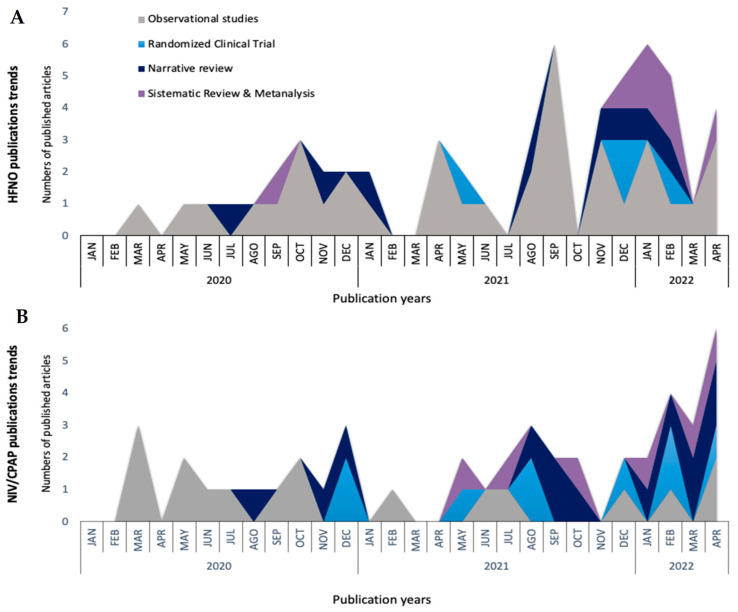
Non-invasive respiratory support publication trends through the COVID-19 pandemic. High-flow nasal oxygen (HFNO) (**A**), non-invasive mechanical ventilation (NIV), and continuous positive airway pressure (CPAP) (**B**).

After the onset of the COVID-19 pandemic, there has been an increase in publications of observational studies (Figure 2A). HFNO has been used since the start of the COVID-19 pandemic as a supportive strategy for COVID-19 patients, as reported in the first Chinese retrospective observational study published at the end of March 2020 [35]. Despite initial concerns about infecting caregivers through aerosolization, the study found that HFNO was the most commonly used first-line respiratory support for patients with COVID-19 pneumonia, being used in 63% of cases [35]. 

From March to December 2020, several observational studies were conducted to examine the use of HFNO in COVID-19 patients [42,43,44,45]. These studies reported intubation rates ranging from 28 to 55% and mortality rates ranging from 14 to 26% [42,43,44,45]. An Italian multicenter cohort study, which involved 670 COVID-19 patients treated with NIRS, reported that HFNO was used in 24% of patients after adjusting for potential confounding factors. The 30-day mortality rates were not significantly different among NIRS groups (HFNO, CPAP, and NIV) [46]. One of the largest retrospective observational studies, performed in 2020 in France enrolled 379 patients and compared those supported with HFNO to patients who did not receive HFNO (treated with standard oxygen therapy, CPAP or NIV). The study found that HFNO significantly reduced the need for intubation and subsequent invasive mechanical ventilation compared to no HFNO use [56% (82/146) vs. 75% (175/233), *p* < 0.001], while mortality rates did not differ between the two groups [21% (30/146) vs. 30% (70/233), *p* = 0.055] [47]. At the beginning of 2021, a multicenter Spanish cohort study compared 61 COVID-19 patients who received HFNO from the first day of ICU admission to 61 patients who were intubated on the first day of admission in ICU [48]. The results showed that the use of HFNO was associated with an increase in ventilator-free days at 28 days (mean difference 8 days; 95% confidence interval [CI] 4.4 to 11.7 days) and a reduction in ICU length of stay (mean difference −8.2 days; 95% CI −12.7 to −3.6 days) compared to early intubation group [48]. Even when most of the baseline characteristics were balanced between the two groups, the early intubation group was composed of more severe patients and the criteria for intubation were not uniformly defined [48]. Another multicenter observational retrospective study conducted in France (n = 245 patients) compared an early intubation group (invasive mechanical ventilation within the first two days after ICU admission) to a NIRS (patients supported with NIV, HFNO, CPAP and oxygen via non-rebreathing mask) [32]. In the NIRS group, 35% of patients received HFNO. The results showed that early intubation was associated with an increased risk of 60-day mortality, but the severity at admission was higher in the early intubation group, and the decision to intubate was therefore not standardized [32]. As formerly reported in patients with hypoxic respiratory failure not related to COVID-19 [40], the use of HFNO may delay intubation. Data also suggests that a prolonged duration of NIRS in respiratory failure may be associated with worse lung damage and an increased risk of requiring extracorporeal membrane oxygenation [49]. 

In September 2021, a Korean observational study reported that late failure of HFNO, defined as the need for invasive mechanical ventilation after 48 h of treatment, was associated with higher mortality in COVID-19 patients compared to early failure (within 48 h) [50]. However, other studies have reported discrepancies with this conclusion, showing that the duration of HFNO prior to intubation does not influence clinical outcomes [51]. Several observational studies have also emphasized the importance of the ROX index, confirming its predictive capacity in COVID-19 patients, especially 6 h after HFNO initiation [51,52,53,54], even though other risk stratification models predicting HFNO failure have been proposed [55,56,57]. At the end of 2021, a large multicenter prospective cohort study conducted in 137 European ICUs found that compared to other types of NIRS, HFNO did not reduce 90-day mortality but was associated with a reduction in NIRS failure (defined as either intubation or death in the ICU without intubation) [58].

**Figure 3:**
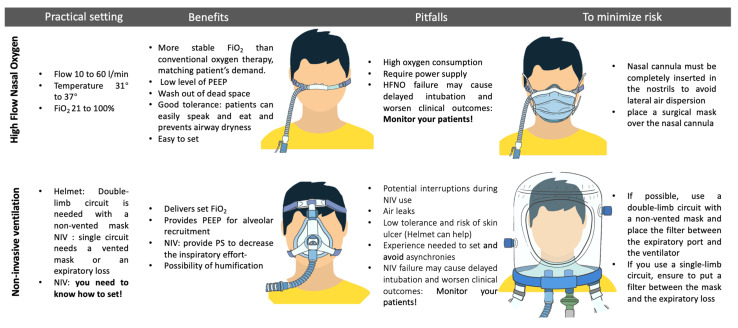
Benefits, risks, and strategies to minimize contamination using non-invasive respiratory support. Inspired fraction of oxygen (FiO_2_); high-flow nasal oxygen (HFNO); pressure support (PS); non-invasive ventilation (NIV); continuous positive airway pressure (CPAP); personal protection elements (PPE); positive end-expiratory pressure (PEEP).

Despite these encouraging results, concerns have been raised about the risk of viral aerosolization and spread [59]. Several studies have been conducted to examine aerosol dispersion and have shown that if HFNO cannulae are placed correctly (completely inserted into the nostrils), the dispersion of HFNO should be lower than the one of standard oxygen via nasal cannulae, oronasal, and venturi mask (17 cm vs. 100 cm, 40 cm, and 33 cm, respectively) [60]. However, since these measurements were obtained from studies performed using human mannequins or healthy volunteers using a surrogate of air, such as smoke, the debate regarding this issue remains unresolved [61,62,63]. Despite the ongoing scientific debate and different recommendations from various scientific societies (Table 1), a consensus has been reached to apply surgical masks on the patients undergoing HFNO treatment and to ensure the proper positioning of the nasal cannulas to reduce the risk of airborne transmission [64,65]. A retrospective evaluation of healthcare worker infections and a multicenter survey found no increase in COVID-19 infection after the implementation of HFNO [66,67]. In both cases, N95 masks were available to healthcare workers. A prospective observational study analyzing the environmental viral contamination of a COVID-19 patient’s room found that either CPAP or HFNO did not result in significant additional air or surface contamination compared to supplemental oxygen [68]. Figure 3 summarizes the main strategies to reduce the risk of contamination. 

In conclusion, practical measures such as proper personal protective equipment, correct placement of nasal cannulae, and the low rate of viral contamination reported in observational studies should encourage more confident use of HFNO. HFNO use in clinical practice has also been supported by cohort studies, which report a reduced need for intubation and subsequent invasive mechanical ventilation with no apparent effect on mortality.

### 3.3. Third Act: Randomized Controlled Trials and Recommendations on HFNO in COVID-19 Patients

Table 2 summarizes the RCTs available as of January 2022 about the use of HFNO in COVID-19 patients. 

The first RCT comparing the use of HFNO to NIV in COVID-19 patients came from an Italian multicenter experience (HENIVOT trial) and was published in May 2021 [36]. The study enrolled 110 patients with acute hypoxemic respiratory failure (PaO_2_/FiO_2_ < 200), who were randomized to receive either helmet NIV or HFNO [36]. No statistical difference was found in the primary endpoint which was the number of days free of respiratory support within 28 days. However, the rate of intubation was significantly lower in the helmet group (30%) than in the HFNO group (51%) (difference, −21% [95% CI, −38% to −3%]; *p* = 0.03) [36]. One limitation of this study was that the reported use of helmet non-invasive ventilation was applied continuously for at least 48 h with high PEEP and that different settings may not provide the same results [36]. In addition, helmet NIV requires expertise, a skilled team, and adequate monitoring [36,69,70]. 

As of December 2021, two other RCTs were published, one comparing HFNO to NIV and the other comparing HFNO to standard oxygen therapy [71,72]. The first was a single-center RCT conducted in India, involving 109 patients who were randomized to receive either HFNO or NIV. The study found that the intubation rate at day 7 was lower in the HFNO group (27%) compared to the NIV group (46%) (relative risk 0.59, 95% CI 0.35–0.99, *p* = 0.045) [71]. In-hospital mortality was similar between the HFNO group (29%) and the NIV group (46%) (relative risk 0.6, 95% CI 0.38–1.04, *p* = 0.06) [71]. The second RCT, HiFLO-COVID, was a multicenter trial conducted across three Colombian hospitals and included 220 COVID-19 patients with PaO_2_/FiO_2_ < 200 and clinical signs of respiratory distress (use of accessory muscles and high respiratory rate) [72]. The patients were randomized to receive either HFNO or conventional oxygen therapy, which was administered via a nasal cannula, venturi mask, or non-rebreathing mask. This study reported that HFNO reduced both the intubation rate (34% vs. 51% hazard ratio 0.62; 95% CI, 0.39–0.96; *p* = 0.03) and time to clinical recovery compared to standard oxygen therapy, but had no effect on mortality or length of ICU stay [72]. 

**Table 2:** Characteristics of RCT investigating HFNO use in patients with COVID-19 acute hypoxemic respiratory failure.

RCT	Country	Population (*n*)	Comparison Group	Primary Outcome	Intubation Rate	Mortality
HENIVOT trial [36]	Italy	110	helmet CPAP	Free days of respiratory support within 28 days.	At day 28: 30% in the CPAP group and 51% in the HFNO group (difference, −21% [95% CI, −38 % to −3%]; *p* = 0.03).	At day 28: 15% in the CPAP group and 18% in the HFNO (absolute difference, −3% [95% CI, −17%to 11%]; *p* = 0.80).
Comparison of HFNC and NIV in Acute Hypoxemic Respiratory Failure Due to Severe COVID-19 Pneumonia [71]	India	109	NIV	Intubation by 48 h.	At day 7: 28% in HFNO group and 46% in NIV group (relative risk 0.59, 95% CI 0.35–0.99, *p* = 0.045).	In-hospital mortality rate was 29.1% in HFNO group and 46.2% in NIV group (relative risk 0.6, 95% CI 0.38–1.04, *p* = 0.06).
HiFLO-COVID [72]	Colombia	220	conventional oxygen therapy	Need for intubation and time to clinical recovery until day 28.	At day 28: 34% in HFNO group and 51% in conventional oxygen group (hazard ratio, 0.62; 95% CI, 0.39–0.96; *p* = 0.03).	At day 28: 8.1% in the HFNO group and 16% in conventional oxygen group (absolute difference, −7.9% [95%CI, −16.9%to 1.1%; hazard ratio 0,49; 95% CI 0.21–1.16]; *p* = 0.11.
RECOVERY-RS trial [73]	United Kingdom	1272	conventional oxygen therapy	Composite of tracheal intubation or mortality within 30 days.	At day 30: 41% in HFNO group and 42% in conventional oxygen group (absolute difference −1% [95% CI, −8%to 6%]; *p* = 0.72).	At day 30: 18.8 in HFNO group and 20% in conventional oxygen group (absolute difference −1% [95% CI, −7% to 4%]; *p* = 0.9).
COVIDICUS Trial [74]	France	333	conventional oxygen therapy and CPAP	Cumulative incidence of invasive mechanical ventilation criteria fulfillment at day 28.	At day 28: 41% of standard oxygen group, 43% of CPAP group, and 44% of HFNO (cause-specific hazard ratio, 1.04 [95% CI, 0.69 to 1.55]; *p* = 0.85).	Sixty-day overall survival was 74% in the HFNO group, 71% in conventional oxygen group, and 72% in CPAP group (mean difference HFNO vs. conventional oxygen 3% [95% CI 2% to 4%]).
SOHO-COVID RCT [75]	France	711	conventional oxygen therapy	Mortality at day 28.	At day 28: 45% in HFNO group and 53% in conventional oxygen group (absolute difference, –7.7% [95% CI, –14.9% to –0.4%]; *p* = 0.04).	At day 28: 10% in HFNO group and 11% in conventional oxygen group (absolute difference, –1.2% [95% CI, –5.8% to 3.4%]; *p* = 0.60).

Helmet Noninvasive Ventilation Versus High-Flow Oxygen Therapy in Acute Hypoxemic Respiratory Failure: HENIVOT trial; High Flow Nasal Oxygen: HFNC; non-invasive ventilation: NIV; High-Flow Nasal Cannula in Severe COVID-19 With Acute Hypoxemic Respiratory Failure: HiFLO-COVID; Efect of noninvasive respiratory strategies on intubation or mortality among patients with acute hypoxemic respiratory failure and COVID-19, a randomized clinical trial: RECOVERY-RS trial; High-Dose Dexamethasone and Oxygen Support Strategies in Intensive Care Unit Patients With Severe COVID-19 Acute Hypoxemic Respiratory Failure, a randomized clinical trial: COVIDICIUS Trial.

The largest RCT about NIRS, the RECOVERY-RS trial, was published in February 2022 [73]. This multicenter trial was conducted across 48 hospitals in the United Kingdom and included 1272 COVID-19 patients with an oxygen saturation of 94% or less while receiving a FiO_2_ > 40%. These patients were randomized to receive CPAP, HFNO, or conventional oxygen therapy (standard face mask or low-flow nasal cannula) [73]. The primary outcome, a composite outcome of intubation or mortality within 30 days of randomization, occurred in 44% of patients in the HFNO group and 45% of patients in the conventional oxygen therapy group (absolute difference, −1% [95% CI, −8% to 6%], *p* = 0.83) [73]. It is worthy of note that the sample size was not achieved because the trial was stopped due to declining COVID-19 cases and intubation criteria were not standardized [73].

The COVIDICUS trial, a French multicenter RCT with the objective of comparing high-dose dexamethasone to standard of care dexamethasone also randomized eligible patients who were not already intubated to receive standard oxygen therapy (via non-rebreathing face mask), CPAP, or HFNO [74]. The primary endpoint for respiratory support was the cumulative incidence of invasive mechanical ventilation criteria fulfillment at day 28. The invasive mechanical ventilation criteria were satisfied in 41% of patients in the standard oxygen group, 43% of the CPAP group, and 44% of the HFNO group, with no significant difference between groups (cause-specific hazard ratio, 1.04 [95% CI, 0.69 to 1.55]; *p* = 0.85) [74].

Last, in September 2022, another French multicentre RCT including 711 COVID-19 patients with a PaO_2_/FiO_2_ equal to or below 200 while receiving oxygen at a flow rate equal to or more than 10 L/min was published [75]. The patients were randomized to receive either conventional oxygen therapy (non-rebreathing mask, with oxygen flow set at 10 L/min or more), or HFNO. The primary outcome, the mortality rate at day 28, did not differ between the two groups (10% in the HFNO group versus 11% in the conventional oxygen group, absolute difference, −1% [95% CI, −5.8% to 3.4%]; *p* = 0.60). However, the intubation rate by day 28 was lower in the HFNO group (45% in the HFNO group versus 53% in the conventional oxygen group, absolute difference of −8% [95% CI, −15% to −0.4%]; *p* = 0.04; hazard ratio of 0.77 [95% CI, 0.63 to 0.96]; *p* = 0.03) [75].

The differences in the setup, intubation criteria, outcomes and weaning of trial interventions make it challenging to draw definitive conclusions on the use of HFNO in patients with COVID-19 presenting with acute respiratory failure. At least 5 systematic reviews and metanalyses were published from January to April 2022, the majority of which included retrospective and prospective observational studies [33,76,77,78,79]. A meta-analysis comparing HFNO and NIV in COVID-19 patients was published in February 2022 [77]. The authors included nine observational studies and one RCT. They found no difference in intubation rates between HNFO and NIV groups (OR, 1.35; 95% CI, 0.86–2.11; *p* = 0.19), while mortality was lower in the HFNO group (OR, 0.49; 95% CI, 0.39–0.63; *p* < 0.001) [77]. Conversely, another meta-analysis addressing the same question and including three of the six published RCTs about HFNO, concluded that it remains uncertain whether HFNO increases or decreases mortality compared to NIV, according to a serious risk of bias, serious imprecision, and indirectness [78].

Several guidelines have addressed the use of NIRS in adult COVID-19 patients with acute hypoxemic respiratory failure [80,81,82,83,84]. Table 3 summarizes the recommendation for HFNO, CPAP, and NIV. The National Institutes of Health and Surviving Sepsis Campaign guidelines suggest starting HFNO therapy in COVID-19 patients with acute hypoxemic respiratory failure when they have insufficient oxygenation, despite receiving conventional oxygen therapy [81,82]. Conversely, the European Respiratory Society and Australian guidelines consider HFNO to be a viable alternative if CPAP is not available or not well tolerated [80,84]. The World Health Organization does not make a formal recommendation about the comparison of HFNO versus CPAP or NIV due to the uncertainty of the data [83]. Almost all the guidelines suggest close monitoring of patients treated with NIRS. 

In conclusion, while cohort studies have shown promising results, the current level of evidence does not permit definitive conclusions on the use of HFNO in COVID-19 patients with acute respiratory failure. Clinical judgment, the expertise of caregivers, and environmental resources remain the key determinants of the use of HFNO in this setting.

## 4. Noninvasive Ventilation

### 4.1. First Act: A Pre-COVID-19 NIRS Strategy and Its Potential Physiological Impact

NIV has become an unavoidable strategy in acute hypercapnic respiratory failure related to acute exacerbation of chronic obstructive pulmonary disease and/or cardiogenic pulmonary edema. In these two settings, NIV has been shown to reduce the risk of intubation [85,86]. Over the past three decades, the scope of NIV application has been greatly expanded beyond acute hypercapnic respiratory failure. Currently, NIV is applied in various settings such as immunocompromised patients with acute hypoxemic respiratory failure, peri-procedures such as bronchoscopy and bronchoalveolar lavage in hypoxemic patients [87], prevention of reintubation [88], and even after abdominal surgery [89,90]. In selected patients experiencing mild acute hypoxemic respiratory failure, NIV may decrease the rate of intubation, mortality, and nosocomial pneumonia [91]. However, it is particularly limited when managing patients with severe acute respiratory failure and can delay intubation [92]. The severity of illness, a potentially reduced level of consciousness, and poor initial response to NIV are one of the most important predictors of failure in this setting [91,93]. ARDS represents the most severe form of hypoxemic acute respiratory failure and given the great variability among their causes, the use of NIV in this group of patients has been difficult to interpret [93]. No recommendation has been made by the official European Respiratory Society/American Thoracic Society clinical guidelines from 2017 [93]. 

The benefits of NIV come from its ability to deliver ventilatory pressure support into the lungs without the need for an invasive endotracheal airway. This pressure support is achieved by programming two pressure levels: the expiratory pressure (expiratory positive airway pressure [EPAP] or PEEP) and the inspiratory pressure (inspiratory positive airway pressure [IPAP]). When the inspiratory effort is detected, NIV delivers inspiratory assistance pressure support using a decelerated flow, keeping IPAP constant, and allowing clinicians to control and improve ventilation [94]. High inspiratory pressure offloads the patient’s breathing effort, while lower pressure preserves an acceptable alveolar volume and prevents unstable alveoli from collapsing during expiration [95,96]. 

CPAP is a NIRS strategy that is provided via a mask connected to a simple airflow/oxygen source, generating a continuous positive pressure over the airway and lungs [96,97,98]. Despite being used as part of NIV, CPAP should be considered separate from NIV as it does not provide pressure support and is not a ‘true’ ventilation strategy [96,99,100]. However, the use of continuous positive airway pressure (CPAP) along with NIV modes has also been reported [97,98]. This continuous positive intrathoracic pressure recruits collapsed alveolar units and increases functional residual capacity and lung compliance. This leads to improved oxygenation and reduced work of breathing and helps to correct ventilation–perfusion mismatch [101,102,103,104]. Studies have shown that CPAP delivered by helmet interface is safer and more effective than a face mask and is better tolerated over prolonged ventilation periods [69,101].

In patients with COVID-19 acute respiratory failure, NIV and CPAP can have positive effects when the patient selection is appropriate. However, care must be taken to avoid harm derived from delayed intubation and excessive respiratory effort. Studies have shown that NIV and CPAP can reduce significantly esophageal pressure and improve oxygenation without demonstrating a difference in dynamic transpulmonary driving pressure compared to HFNO (9.9 ± 3.8; 7.6 ± 4.3; 8.8 ± 3.6 during HFNO, CPAP, and NIV, respectively) [105]. CPAP may also decrease lung injury from excessive diaphragmatic contraction in dorsal regions by avoiding large transpulmonary pressure swings and tidal volumes, thus preventing excessive Pendelluft phenomenon and P-SILI [105,106].

Despite the high demand for respiratory support and a shortage of resources to manage the increasing number of patients with acute respiratory failure, the use of NIV for COVID-19 patients was not widely adopted. This was mainly due to concerns about delayed intubation, P-SILI virus, and virus transmission through aerosol generation, the latter being a major concern [107]. 

### 4.2. Second Act: The Observational Phase—Insights from Clinical Experience with NIV and CPAP

NIV was used early in the COVID-19 pandemic. The first report of its use was from a retrospective observational study in China (December 2019), which found that 29 of the 52 critically ill adult patients with COVID-19 pneumonia admitted to ICU received NIV (56%) [3]. Sixty-seven percent of these patients had ARDS with the PaO_2_/FiO_2_ ratio ranging from 52 to 126.7 [3]. Thirty percent of NIV users survived, compared to only 15% of those who received invasive support. However, no details were provided regarding the severity of ARDS in the NIV survivor group, the NIV modality used, or the interface used [3]. Similarly, Mukhtar et al. conducted a retrospective study of 55 patients with ARDS admitted to ICU from May 2020 to July 2020 [108]. Of the 39 patients (71%) who required ventilatory support (invasive or not), 30 (77%) avoided intubation thanks to successful NIV. This suggests that NIV was feasible with a reasonable success rate. Importantly, the severity of ARDS according to the PaO_2_/FiO_2_ ratio was similar for those receiving NIV and those receiving invasive mechanical ventilation [108]. 

Nevertheless, the NIV use varied greatly among countries. Only 4% (4 out of 225 patients) of COVID-19 patients admitted to Australian intensive care units in the early pandemic received NIV on their first day at the ICU [109]. Similarly, a German study also reported low use of NIV on the first day of ICU admission, with only 8% (18 of 57 patients using NIRS) receiving NIV. However, 81% of these patients (46 out of 57) required subsequent intubation due to NIV/HFNO therapy failure [110]. The low success rate of NIV could partially explain its limited use in some countries, compared to previous reports [3,108].

Since NIV and CPAP are listed by the World Health Organization as high-risk aerosol-generating procedures, one of the concerns that limited the use of NIV or CPAP in the early reports was the airborne risk for patients and healthcare workers [107,111,112]. To reduce the airborne transmission, it was recommended to fit viral filters to the expiratory limb of the circuit and to use negative pressure single rooms, if available. Non-vented masks that covered the patient’s nose and mouth, such as the helmet, were also used. The helmet was an interface surrounding the patient’s head made of transparent plastic with a soft collar and a double-limb circuit [112]. The growing evidence related to NIV and CPAP administration (Figure 2B) suggests that the helmet is an interesting option if is it available and the team knows how to use it. However, it remains in many cases as an expert’s option [69,101,113].

To ensure the safe use of NIV or CPAP, special units or teams outside the ICU were established. Nightingale et al. reported the use of CPAP to treat hypoxemic respiratory failure due to COVID-19 in negative pressure rooms in a new infectious disease unit [110]. A case series of 24 patients admitted to the Royal Liverpool Hospital between April 1st and April 3rd, 2020 showed that over half of the patients (58%) avoided invasive mechanical ventilation, 1 died on CPAP, and 4 died receiving invasive support [110]. Importantly, the PaO_2_/FiO_2_ ratio ranged from 97 to 175, and the median time to intubation was just 4 h, which contrasts with other studies reporting a median of 1 day until intubation [110]. Delaying intubation due to NIV/CPAP failure became a major concern because it could worsen lung injury caused by PSILI [114]. Patients in a severe stage of disease, with a PaO_2_/FiO_2_ ratio ≤ 100, should not wait for invasive support. Starting NIV/CPAP in patients with ARDS in the moderate stage (PaO_2_/FiO_2_ ratio between 100 and 200) has been associated with a reduction of both in-hospital mortality and hospitalization length compared to the severe stage; meanwhile, starting NIV or CPAP with a PaO_2_/FiO_2_ ratio > 150 will not present any clinical advantage [115].

The use of NIRS, specifically Bilevel-NIV/CPAP, continued to have growing evidence in 2021 with systematic reviews suggesting high heterogeneity among studies. Only 3 out of 17 studies were prospectively conducted, highlighting the need for well-designed clinical trials [46,116,117]. The in-hospital mortality of patients receiving NIV/CPAP outside the ICU was 36% with a 26% failure rate requiring intubation [114]. Although NIV/CPAP is seen as a feasible strategy for addressing the high demand for ventilatory support, more evidence from randomized clinical trials was needed to meet the recommendationn criteria.

### 4.3. Third Act: Randomized Controlled Trials about NIV and CPAP in COVID-19 Patients

The first RCT examining the use of NIV for COVID-19 patients with acute hypoxemic respiratory failure was the HENIVOT trial, published in May 2021 [36]. It involved 110 patients who were randomized to receive either helmet NIV or HFNO. As previously mentioned in the HFNO section, there was no statistical difference found in the number of days free of respiratory support within 28 days, but the rate of intubation was significantly lower in the helmet group (30%) compared to the HFNO group (51%) [36]. A post hoc analysis published by the same authors showed that the physiological benefits of helmet NIV over HFNO were more pronounced in patients with more severe oxygenation impairment and intense inspiratory effort. This suggests that there is a phenotype profile of patients who may have a better response to NIV [118]. The second RCT on NIV, published in 2021, included 109 patients who were also randomized to receive either HFNO or NIV [71]. The results showed that the intubation rate at day 7 was lower in the HFNO group (27%) compared to the NIV group (46%) [71]. However, there were no significant differences in oxygenation parameters, intubation rate at 48 h, or hospital mortality between groups [71]. 

The third randomized clinical trial was the RECOVERY-R, which was published in 2022, and compared CPAP or HFNO with conventional oxygen therapy and was conducted between April 2020 and May 2021. The trial was stopped prematurely due to declining COVID-19 cases, but still included 12,673 patients. The results showed a lower tracheal intubation rate with CPAP (36%; 137 of 377 participants) versus conventional oxygen therapy (44%; 158 of 356 participants), but there was no significant difference with HFNO (44%; 184 of 415 participants) and conventional oxygen therapy (45%; 166 of 368 participants) [73].

A recent randomized clinical trial from September 2022 suggested that helmet NIV did not have a significant impact on reducing 28-day mortality compared to standard respiratory support in patients with acute hypoxemic respiratory failure caused by COVID-19 pneumonia [119]. However, the study has several limitations that need to be considered, such as the short amount of time for centers to be trained in using the helmet, which has a learning curve, and the moderate levels of PEEP used in the helmet NIV group [119]. Helmet NIV is more comfortable and longer-lasting than face mask NIV [69] and was associated with a significant reduction in intubation rates and 90-day mortality [70]. However, as mentioned before, the use of helmet requires specialized skills, a trained team, and proper monitoring, which can hinder its widespread implementation [61].

In summary, according to the guidelines (Table 3), CPAP/NIV delivered through either a helmet or a facemask is a suitable option for patients with COVID-19 and hypoxemic acute respiratory failure who do not require immediate intubation [80,120]. Although RCTs show that CPAP may offer an advantage over HFNO, and that NIV could offer some advantages in patients with more severe oxygenation impairment and intense inspiratory effort, more well-designed RCT are required to establish the optimal use of NIV/CPAP in this patient population.

## 5. Conclusions

NIRS gained importance during the COVID-19 pandemic due to its reduced risk of complications and its ability to alleviate ICU overcrowding. Although the use of HFNO has shown a reduced need for intubation and subsequent invasive mechanical ventilation with no apparent effect on mortality, CPAP (delivered through either a helmet or a facemask) may offer some clinical benefits over HFNO, and NIV could offer some advantages in patients with more severe oxygenation impairment and intense inspiratory effort.

Clinical judgment, caregiver expertise, and resource availability remain key determinants in the use of NIRS in treating COVID-19 patients with acute respiratory failure. Research on NIRS in COVID-19 acute respiratory failure has dramatically increased, but further confirmation of its benefits and potential complications through larger trials is still needed, especially due to concerns about delayed intubation, virus transmission, and P-SILI.

## Figures and Tables

**Table 1:** What guidelines report about airborne transmission while using HFNO in COVID-19 patients.

Guidelines	What They Report about HFNO and Risk of Contamination
European respiratory society (ERS) Clinical Practice Guidelines	Use of HFNO requires protective personal equipment and ventilation (unknown risks of transmissibility).
National Insistutes of Health (NIH) COVID Treatment Guideline	It remains unclear whether the use of HFNO oxygen results in a lower risk of nosocomial SARS-CoV-2 transmission than NIV.
Surviving Sepsis Campaign Guidelines	HFNO does not seem to confer an increased risk of transmission of disease.
World Health Organization (WHO): Management of critical COVID-19 Advanced non-invasive respiratory support	A respirator should always be worn (very low certainty evidence) along with other personal protective equipment (including gown, gloves, eye protection) when treating patients with HFNO.
Australian Guidelines	Considering the aerosol risk to staff and other patients, the preferred location for HFNO is a negative pressure room or a single room with the door closed.

**Table 3:** Summary of recommendations about the use of NIRS in adult COVID-19 patients with acute hypoxemic respiratory failure.

Guidelines	HFNO	CPAP and NIV
European Respiratory Society living guideline	The panel suggests HFNO for patients without an immediate need for invasive mechanical ventilation and who are unsuitable for CPAP due to intolerance or adverse effects (conditional recommendation).	The panel suggests CPAP (via helmet or facemask) for patients without an immediate need for invasive mechanical ventilation (conditional recommendation).
National Insistutes of Health-COVID treatment guideline	For COVID-19 patients with acute hypoxemic respiratory failure despite conventional oxygen therapy, the panel recommends starting therapy with HFNO (BIIa).	If HFNO is not available and the patient does not have an indication for endotracheal intubation, the panel recommends performing a closely monitored trial of NIV (BIIa). If patients fail to respond to HFNO, NIV or intubation and mechanical ventilation should be initiated (BIIa).
Surviving Sepsis Campaign guidelines	For COVID-19 with acute hypoxemic respiratory failure despite conventional oxygen therapy, we suggest using HFNO (weak). In adults with COVID-19 and acute hypoxemic respiratory failure, we suggest HFNO over NIV (weak). Close monitoring	In COVID-19 adults with acute hypoxemic respiratory failure, if HFNC is not available and there is no urgent indication for endotracheal intubation, we suggest a trial of NIV with close monitoring (weak).
World Health Organization: clinical management of COVID-19	In patients with COVID-19 and acute hypoxemic respiratory failure not needing emergent intubation, the panel suggests HFNO, CPAP or NIV rather than standard oxygen therapy (conditional recommendation). Clinicians should choose between different NIRS according to availability, experience, and patient-specific considerations.	
Australian guidelines for the clinical care of people with COVID-19	If CPAP is not available or not tolerated, consider HFNO as an alternative to conventional oxygen delivery (conditional recommendation). Close monitoring.	For COVID-19 patients with acute hypoxemic respiratory failure despite conventional oxygen therapy, consider using CPAP (conditional recommendation). There is currently insufficient direct evidence available to support the use of NIV.

## Data Availability

Not applicable.
